# Stress and Sleep Dynamics in Young Adults with Psychotic-Like Experiences Across Daily Life

**DOI:** 10.1093/schbul/sbag104

**Published:** 2026-08-29

**Authors:** Błażej Misiak, Paulina Bagrowska, Jerzy Samochowiec, Łukasz Gawęda

**Affiliations:** Department of Psychiatry, Wroclaw Medical University, 50-367 Wroclaw, Poland; Experimental Psychopathology Lab, Institute of Psychology, Polish Academy of Sciences, 00-378 Warsaw, Poland; Department of Psychiatry, Pomeranian Medical University, 71-460 Szczecin, Poland; Experimental Psychopathology Lab, Institute of Psychology, Polish Academy of Sciences, 00-378 Warsaw, Poland

**Keywords:** insomnia, psychosis, early-life stress, stress sensitivity, ecological momentary assessment

## Abstract

**Background and hypothesis:**

Psychotic-like experiences (PLEs) confer increased risk for adverse mental health outcomes. Sleep disturbances are associated with PLEs, yet evidence largely relies on retrospective assessments and average sleep parameters, limiting insight into daily-life dynamics. Moreover, the roles of stress exposure in sleep–PLEs associations remain insufficiently understood. This study aimed to address these gaps by examining sleep characteristics, momentary stress, and childhood trauma in relation to PLEs using experience sampling. We hypothesized that individuals with PLEs show poorer and more variable sleep, bidirectional sleep–PLEs associations, stronger stress-related sleep disturbances, and trauma-related alterations in sleep regulation.

**Study Design:**

Altogether, 99 individuals with PLEs and 102 controls completed the experience sampling across 7 consecutive days.

**Study Results:**

Individuals with PLEs reported poorer average sleep quality, shorter sleep duration, and greater variability in sleep quality, although differences in sleep quality and its variability were attenuated after adjustment for negative affect. Within the PLEs group, sleep quality, but not sleep duration, showed robust bidirectional within-person associations with PLEs. Daily stress fluctuations were not associated with next-morning sleep, whereas higher between-person stress exposure was related to poorer average sleep quality, with no moderation by PLEs group status. Childhood trauma was not significantly associated with sleep outcomes.

**Conclusions:**

Reduced sleep duration might be a stable feature of PLEs independent of negative affect, while sleep quality is dynamically linked to momentary PLEs. Sleep disturbances in individuals with PLEs seem more closely related to symptom dynamics than to momentary stress and distal developmental risk factors.

## Introduction

Psychotic-like experiences (PLEs) are subclinical psychosis-spectrum phenomena, such as perceptual abnormalities and unusual beliefs, occurring without a diagnosed psychotic disorder.^1^ They are conceptualized within the framework of the extended psychosis phenotype, which assumes variability in expression, persistence, and clinical significance across the general population.^2^ Epidemiological studies indicate that PLEs are common, especially in adolescents and young adults.^2^^,^^3^ Moreover, PLEs are associated with elevated psychological distress, mood and anxiety disorders, functional impairment, increased mental health service use,^4–6^ and prospectively predict adverse outcomes.^7–9^ Although their prognostic relevance depends partly on persistence and associated distress,^10^ even non-distressing PLEs may confer elevated risk.^11^ Importantly, reliance solely on self-report screening may inflate prevalence and false-positive classification, underscoring the need for clinically informed assessment.^12^^,^^13^

There is evidence that PLEs are embedded in broader disturbances of emotional and physiological regulation,^14–17^ processes in which sleep plays a central role.^18^ Sleep disturbances are widely documented across the psychosis spectrum and in at-risk populations.^19^ Shorter sleep duration has been bidirectionally associated with PLEs,^20–22^ and experimental studies suggest that sleep loss may play a causal role.^23^ Experience sampling method (ESM) studies—an intensive longitudinal approach capturing momentary experiences in daily life^24^—have further shown that poorer sleep quality and shorter sleep duration predict subsequent increases in psychotic symptoms and PLEs.^25–34^ Some of these associations might be mediated by negative affect.^31–34^ However, evidence points to partial bidirectionality, as psychotic symptoms might also predict subsequent sleep quality, albeit with smaller effect sizes.^31^ Beyond average sleep characteristics, day-to-day variability in sleep timing and quality may represent a marker of regulatory instability, as greater variability has been linked to affective dysregulation, stress sensitivity, and adverse health outcomes, independent of mean sleep duration or quality.^35^^,^^36^ In the context of psychosis-spectrum vulnerability, such unstable sleep patterns may reflect dynamic regulatory impairment unfolding in daily life rather than persistent deficits in average sleep parameters.

Both sleep disturbances and negative affect are sensitive to fluctuations in daily stress, suggesting that stress-related processes may play a critical role in shaping sleep–PLEs dynamics. Stress exposure has long been implicated in psychosis-spectrum vulnerability, with evidence indicating that individuals with PLEs exhibit heightened emotional and psychotic reactivity to daily stressors.^14^^,^^16^^,^^37^^,^^38^ However, it remains unknown whether increased stress sensitivity extends to sleep disturbances.

It has also been shown that childhood trauma may exert enduring effects on emotional and physiological adaptation, manifesting in persistent alterations in emotion regulation, heightened threat sensitivity, and long-term changes in neurobiological stress systems.^39–43^ These developmental adaptations may confer a more enduring vulnerability profile characterized by increased affective instability and altered regulation of physiological states. Childhood trauma has been linked to poorer subjective sleep quality, increased sleep fragmentation, insomnia symptoms, and disruptions in sleep continuity across both clinical and non-clinical populations.^42^^,^^43^ Such associations may reflect enduring dysregulation of stress and arousal systems that confer transdiagnostic vulnerability. However, it remains unclear whether trauma-related sleep disturbances differ as a function of psychosis-spectrum liability or whether they contribute to sleep instability that may, in turn, shape PLEs expression.

To address research gaps, we examined sleep disturbances in the context of psychosis-spectrum vulnerability across multiple levels of analysis. Consistent with the extended psychosis phenotype framework, we compared individuals with versus without PLEs using a clinically informed classification to capture meaningful variation in psychosis liability. We conceptualized sleep disturbances within a multilevel stress-vulnerability framework, distinguishing between relatively stable distal and between-person vulnerability markers and dynamic (within-person), daily-life processes. Our hypothesis was that individuals with PLEs would exhibit poorer and more variable sleep quality and shorter sleep duration. We further hypothesized bidirectional associations between daily fluctuations in sleep and momentary PLEs. Based on stress-reactivity models,^16^^,^^37^ we expected that daily stress would predict poorer subsequent sleep outcomes, particularly among individuals with PLEs, reflecting heightened stress sensitivity in the sleep domain. Finally, we hypothesized that childhood trauma, a distal indicator of long-term stress exposure, would be associated with person-level sleep parameters, with stronger associations among individuals with PLEs.

## Methods

### Recruitment Procedures

Detailed information about recruitment procedures can be found elsewhere.^14^^,^^44^ Participant recruitment followed a staged procedure based on a sequential extreme-groups strategy with interview confirmation (Figure S1). Briefly, individuals with the highest screening scores were recruited as potential members of the PLEs group and underwent clinical verification using Comprehensive Assessment of At-Risk Mental States (CAARMS), whereas individuals with the lowest screening scores were recruited as controls without PLEs. Initially, an online screening survey was conducted to identify individuals reporting PLEs. Eligibility for completing the screening survey required participants to be aged 18-35 years, have no lifetime history of psychiatric treatment, and reside within the catchment areas of the participating research centers (Szczecin, Warsaw, or Wroclaw in Poland). The study was limited to drug-naïve participants to focus on early manifestations of psychosis-spectrum vulnerability prior to clinically treated psychiatric disorders and to minimize potential confounding effects of psychotropic medications on sleep. Results from this screening phase have been reported previously.^45–47^

The screening questionnaire comprised 16 items assessing PLEs experienced during the preceding month. These items were adapted from the Revised Hallucination Scale,^48–50^ the Revised Green et al. Paranoid Thoughts Scale,^51^^,^^52^ and the Prodromal Questionnaire–16.^52^^,^^53^ Responses were recorded on a 4-point Likert scale ranging from 1 (“never”) to 4 (“almost always”), yielding total scores between 16 and 64. In addition, screening items from the Mini-International Neuropsychiatric Interview (MINI)^54^ were included. Participants were ranked according to their total screening scores of PLEs, and recruitment proceeded sequentially from both ends of the distribution. Individuals from the upper end of the distribution were contacted as potential members of the PLEs group, whereas individuals from the lower end were approached as potential control participants. No predefined cut-off scores were used for group assignment.

In the subsequent stage, individuals with the highest screening scores were contacted for telephone interviews. The presence of PLEs was evaluated using selected items from the CAARMS,^55^^,^^56^ focusing on ideas of reference, suspiciousness/persecutory ideation, and hallucinations rated on the global severity scale (0 = “absent” to 6 = “severe and psychotic”). The CAARMS assessment served as the clinical verification step to determine final group assignment and to reduce potential false-positive classification based on self-report screening measures. Participants who endorsed screening items indicating possible current major depressive episodes or substance use problems were further assessed using the relevant diagnostic modules of the MINI to determine whether they met exclusion criteria. Eligibility for the in-person assessment phase required meeting the original age and treatment history criteria and scoring between 3 and 5 on CAARMS global ratings for ideas of reference and/or suspiciousness/persecutory ideation, and/or between 3 and 4 for hallucinations, corresponding to attenuated psychotic symptoms. Exclusion criteria included any lifetime psychiatric treatment, a current severe major depressive episode, or a diagnosis of substance use disorder other than nicotine dependence. In parallel, individuals with the lowest screening scores of PLEs were contacted via telephone to recruit a control group without PLEs. Recruitment within each group continued sequentially until a target sample size of approximately 100 participants per group was reached, reflecting practical constraints associated with interview-based screening procedures and the demands of intensive longitudinal data collection.

During the final recruitment stage, participants completed full versions of the MINI and CAARMS and were subsequently enrolled in the ESM protocol. The study received ethical approval from the Ethics Committees of the Institute of Psychology, Polish Academy of Sciences in Warsaw (approval no. 16/VII/2022), Wroclaw Medical University (approval no. 129/2022), and Pomeranian Medical University in Szczecin (approval no. KB-006/25/2022). Written informed consent was obtained from all participants prior to study participation.

### Childhood Trauma

A history of childhood trauma was assessed using the Traumatic Experiences Checklist (TEC).^57^ The TEC assesses exposure to 29 potentially traumatic experiences occurring before the age of 18 and records information across 3 subscales: presence of the event (present vs. absent), age at exposure, and subjective impact rated on a 5-point scale (1 = “none” to 5 = “an extreme amount”). In the present study, analyses focused on 4 widely studied categories of childhood trauma: emotional abuse, emotional neglect, physical abuse, and sexual abuse. According to the proposed scoring algorithm, for each trauma category, exposure is evaluated across 3 developmental periods (0-6 years, 7-12 years, and 13-18 years). Within each developmental period, 4 characteristics are assessed: (1) presence of the trauma (0 = absent, 1 = present); (2) duration (0 = up to 1 year, 1 = longer than 1 year); (3) relationship to the perpetrator (0 = non-family or extended family, 1 = parents or siblings); and (4) subjective impact (0 = ratings of 1-2, 1 = ratings of 3-5). Scores for each trauma category are calculated by summing these indicators across developmental periods, yielding a total score ranging from 0 to 12, with higher scores indicating greater exposure to that specific type of childhood trauma.

### ESM

Participants completed assessments over 7 consecutive days using a semi-random prompting schedule. Six assessments per day were delivered via push notifications through the *MovisensXS* mobile application between 9:00 am and 10:00 pm. Sleep quality and duration were assessed once per day, during the first daily assessment. The application allowed offline completion of questionnaires, with responses stored locally and uploaded automatically once an internet connection was available. All entries were automatically time-stamped, enabling monitoring of compliance and exclusion of delayed or missed responses. Prior to commencing the protocol, participants received standardized instructions regarding the use of the application and study procedures. For analytic purposes, responses were considered valid only if submitted within 15 min of the notification; entries outside this window were excluded. Participants received gift vouchers (equivalent to 250 EUR) contingent on achieving a response rate of at least 80%, a criterion met by all participants.

The ESM questionnaires comprised between 39 and 42 items per assessment. Analyses in the present study focused on items assessing PLEs, negative affect, sleep quality and duration, and multiple domains of psychosocial stress (see Table S1 for item wording and scoring). The items referring to sleep (quality and duration) and the item assessing event-related stress referring to the preceding 2 h (“Think about the most important event during the last two hours. This event was…”) were administered only during the first morning assessment. Completion of a single questionnaire required approximately 2-3 min (mean completion time: 2 min 21 s). Internal consistency of multi-item scales was acceptable-to-excellent, as indicated by Cronbach’s alpha coefficients (Table S1).

### Data Analysis

Preprocessing and data analyses were carried out in R (version 4.4.0). Before data analyses, some items were reverse coded and the mean scores for multi-item constructs were estimated (Table S1). For descriptive purposes, participants with PLEs and controls were compared with respect to general characteristics using the χ^2^ test (categorical variables) and *t*-tests or Mann–Whitney U test (continuous variables; depending on data distribution).

To examine group differences and trauma associations with overall sleep characteristics, sleep outcomes were aggregated at the person level. For each participant, we computed mean sleep quality, mean sleep duration, sleep quality variability (within-person SD), and sleep duration variability (within-person SD). Linear regression models were used to examine associations of these sleep outcomes with group status (individuals with PLEs vs. controls), childhood trauma dimensions (emotional neglect, emotional abuse, physical abuse, and sexual abuse), and group status by childhood trauma interactions. Because group status and childhood trauma variables were time-invariant, all trauma analyses were conducted at the person level. False discovery rate (FDR) correction was applied separately within each family of tests (mean sleep outcomes and variability outcomes), correcting across trauma categories for each outcome type.

To examine temporal associations between sleep and PLEs in daily life, we used day-level sleep reports obtained at the first morning assessment of each day (sleep duration and sleep quality referring to the preceding night). These variables were merged with momentary PLEs ratings collected across beeps during the same day. Bidirectional models were estimated. First, same-day associations were examined by predicting momentary PLEs from nightly sleep duration and sleep quality. Second, next-day associations were examined by predicting next-morning sleep outcomes from the previous day’s mean PLE level. Time-varying predictors were decomposed into within-person and between-person components using person-mean centering, separating day-to-day deviations from stable individual differences. Linear mixed-effects models included random intercepts for participants. FDR correction was applied to the primary temporal association tests.

To examine stress sensitivity, linear mixed-effects models were estimated predicting next-morning sleep outcomes (sleep quality and sleep duration) from previous-day stressors. Stressors included event-related stress, area-related stress, social stress, and activity-related stress. Each stressor was decomposed into within-person and between-person components. The within-person component represented day-to-day deviations from an individual’s own mean stress level. The between-person component was the individual’s average stress level across the sampling period. Models included random intercepts for participants. Fixed effects were within-person stress, between-person stress, group status (individuals with PLEs vs. controls), and interactions (within-person stress × group status and between-person stress × group status). *P*-values for interaction effects were corrected for multiple testing using the FDR procedure, applied only to interaction terms, as these represented the primary hypotheses regarding group differences in stress sensitivity.

Statistical models were adjusted for age, sex, and education (adjustment 1). In addition, negative affect was included as a covariate to account for the potential confounding influence of general emotional distress, which is strongly associated with sleep disturbances, stress exposure, and PLEs (adjustment 2).^16^^,^^31^^,^^32^ Adjusting for negative affect was intended to examine associations independent of concurrent affective states rather than to model causal pathways. In mixed-effects models, negative affect was decomposed into within-person and between-person components using the same person-mean centering procedure as applied to other time-varying predictors.

Statistical significance was defined as *P* < .050. Where applicable, FDR-adjusted *P*-values were reported. Given the intensive longitudinal nature of the study, a conventional a priori power calculation was not performed. Instead, simulation-based design sensitivity analyses, which are recommended for multilevel ESM data,^58^^,^^59^ were performed in the *simr* package.^60^ These simulations account for both the number of participants and the number of within-person observations, providing a more realistic evaluation of detectable effect sizes in clustered data. The simulations assumed a 2-level structure (observations nested within individuals) and were parameterized to match the present study design, including 201 participants (99 with PLEs and 102 controls), approximately 40 valid assessments per participant, intraclass correlation coefficients ranging from 0.2 to 0.5, and standardized predictors and outcomes. The design sensitivity analysis focused on group × stress interactions, as these represent the most statistically demanding effects tested in the present study. For person-level regression models examining childhood trauma, power calculations were performed analytically based on Cohen’s f^2^ effect size metric in the *pwr* package.^61^

## Results

### General Characteristics of the Sample

Altogether, 99 individuals with PLEs and 102 controls completed the ESM protocol (Table 1). They were enrolled from the sample of 4203 individuals (aged 25.3 ± 5.7, 63.8% women) screened for PLEs (Figure S1). Participants with PLEs were significantly younger and had higher lifetime rates of any mood and anxiety disorders. As expected, they had higher scores of self-reported and clinically-rated PLEs. The scores of emotional abuse, emotional neglect, and sexual abuse, but not physical abuse, were significantly higher among participants with PLEs. Both groups had a comparable response rate in the ESM protocol. The levels of daily stress across all categories, negative affect, and PLEs were significantly higher in participants with PLEs compared to controls.

**Table 1:** General Characteristics of Participants.

	PLEs (+), *n* = 99	PLEs (−), *n* = 102	*P*
Age, years	23.9 ± 5.0	26.5 ± 5.1	<.001
Sex, M (%)	23 (23.2)	30 (29.4)	.320
**Education** Primary Vocational Secondary Higher	5 (5.0) 0 (0) 48 (48.5) 46 (46.5)	1 (1.0) 1 (1.0) 40 (39.2) 60 (58.8)	.102
Employed or student	92 (92.9)	98 (96.1)	.368
Married or informal relationship	63 (63.6)	74 (72.5)	.175
**Diagnosis—lifetime (MINI)** Mood disorder Anxiety disorder	53 (53.5) 48 (48.5)	24 (23.5) 21 (20.6)	<.001 <.001
PLEs, screening score	32.9 ± 6.2	19.0 ± 4.8	<.001
CAARMS, subclinical positive symptoms^**a**^	7.9 ± 4.0	0.8 ± 1.6	<.001
**Childhood trauma (TEC)** ^**b**^ Emotional abuse Emotional neglect Physical abuse Sexual abuse	5.4 ± 3.7 5.4 ± 4.1 3.4 ± 2.7 1.1 ± 1.7	2.3 ± 3.3 3.2 ± 4.3 3.2 ± 1.9 0.3 ± 0.8	<.001 <.001 .123 <.001
**ESM** Valid questionnaire responses PLEs Negative affect Event-related stress Activity-related stress Area-related stress Social stress	3973 (95.3) 2.0 ± 1.0 3.0 ± 1.5 3.6 ± 1.6 3.5 ± 1.7 2.6 ± 1.8 3.0 ± 1.7	4067 (94.9) 1.1 ± 0.4 1.7 ± 1.0 3.3 ± 1.4 2.9 ± 1.4 2.0 ± 1.5 2.5 ± 1.5	.184 <.001 <.001 <.001 <.001 <.001 <.001

Abbreviations: CAARMS = the Comprehensive Assessment of At-Risk Mental State; ESM = experience sampling method; MINI = the Mini-International Neuropsychiatric Interview; PLEs = psychotic-like experiences; TEC = the Traumatic Experiences Checklist.

^a^The total score across the severity and frequency of positive symptoms across all subscales, ie, unusual thought content, non-bizarre ideas, perceptual abnormalities, and disorganized speech (range: 0-48).

^b^Missing data in 12 participants with PLEs and 9 participants without PLEs.

### Person-Level Sleep Outcomes

**Figure 1:**
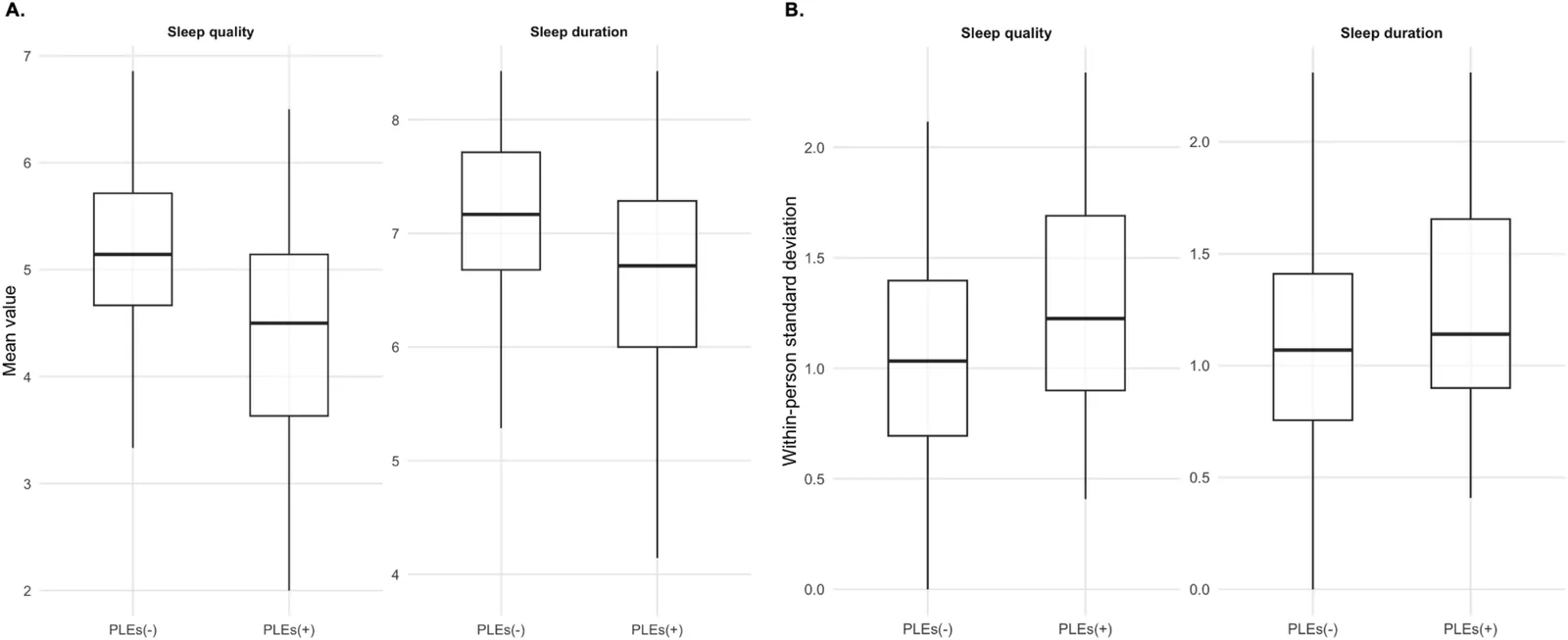
Person-Level Sleep Characteristics in Individuals with and without Psychotic-Like Experiences (PLEs). Panel (A) shows mean sleep quality and mean sleep duration (hours), whereas Panel (B) shows within-person variability in sleep quality and sleep duration, indexed as the SD across the experience-sampling period. Boxplots depict the distributions of person-level estimates for participants with PLEs (PLEs(+)) and those without PLEs (PLEs(−)). Boxes represent interquartile ranges, horizontal lines indicate medians, and whiskers extend to 1.5 × IQR.

Participants with PLEs differed from controls in several person-level sleep parameters (Table 2**,**  Figure 1). In unadjusted analyses, participants with PLEs reported significantly poorer mean sleep quality (b = −0.714, SE = 0.132, *P* < .001) and shorter mean sleep duration (b = −0.568, SE = 0.212, *P* = .008). These differences remained significant after adjustment for age, sex, and educational level. After further adjustment for mean person-level negative affect, the group difference in sleep quality was attenuated and no longer significant (b = −0.252, SE = 0.164, *P* = .126), whereas the difference in sleep duration remained statistically significant (b = −0.675, SE = 0.277, *P* = .031).

**Table 2:** Differences in Person-Level Sleep Outcomes Between Individuals With Psychotic-Like Experiences and Controls.

Sleep outcome	Unadjusted analysis	Adjustment 1	Adjustment 2
Sleep quality (mean)	b = –0.714, SE = 0.132, *P* < .001	b = –0.657, SE = 0.137, *P* < .001	b = –0.252, SE = 0.164, *P* = .126
Sleep duration (mean)	b = –0.568, SE = 0.212, *P* = .008	b = –0.576, SE = 0.221, *P* = .010	b = –0.675, SE = 0.277, *P* = .031
Sleep quality (variability)	b = 0.240, SE = 0.070, *P* = .002	b = 0.227, SE = 0.072, *P* = .003	b = 0.162, SE = 0.090, *P* = .143
Sleep duration (variability)	b = 0.259, SE = 0.392, *P* = .509	b = 0.161, SE = 0.411, *P* = .695	b = –0.306, SE = 0.513, *P* = .552

Adjustment 1 includes the effects of age, sex, and the level of education. Adjustment 2 includes the effects of age, sex, the level of education, and mean person-level negative affect. FDR-adjusted *P*-values are reported.

With respect to sleep variability, individuals with PLEs showed greater variability in sleep quality compared with controls in unadjusted analyses (b = 0.240, SE = 0.070, *P* = .002) and after adjustment for age, sex, and education (b = 0.227, SE = 0.072, *P* = .003). However, this association was no longer significant after controlling for mean negative affect (b = 0.162, SE = 0.090, *P* = .143). No significant group differences were observed for variability in sleep duration in any of the models (all *P* > .050).

The analyses performed in participants with PLEs revealed no significant between-person associations between sleep outcomes and the level of PLEs (Table S2).

### Directional Associations of Sleep Outcomes with PLEs

Within the group of individuals with PLEs, directional within- and between-person associations between sleep parameters and PLEs are reported in Table 3. A significant bidirectional association at the within-person level was found between PLEs and sleep quality. Specifically, higher levels of PLEs predicted poorer sleep quality the following night in unadjusted analyses (b = –0.425, SE = 0.156, *P* = .026) and after adjustment for age, sex, and education (b = –0.424, SE = 0.156, *P* = .026). This association remained significant after additional adjustment for negative affect (b = –0.502, SE = 0.174, *P* = .031). Conversely, poorer sleep quality predicted higher levels of PLEs during the following day in unadjusted analyses (b = –0.037, SE = 0.008, *P* < .001) and after accounting for age, sex, and education (b = –0.037, SE = 0.008, *P* < .001). This association also remained significant after additional adjustment for negative affect (b = –0.018, SE = 0.008, *P* = .028).

**Table 3:** Directional Associations of Psychotic-Like Experiences (PLEs) With Sleep Quality and Duration.

Path	Unadjusted analysis			Adjustment 1			Adjustment 2		
	b	SE	*P*	b	SE	*P*	b	SE	*P*
PLEs **→** sleep quality (WP)	–0.425	0.156	.026	–0.424	0.156	.026	–0.502	0.174	.031
PLEs **→** sleep quality (BP)	–0.287	0.146	.098	0.266	0.149	.147	–0.088	0.178	.776
PLEs **→** sleep duration (WP)	–0.185	0.371	.619	–0.190	0.371	.695	–0.204	0.414	.776
PLEs **→** sleep duration (BP)	–0.114	0.229	.619	–0.072	0.230	.754	–0.040	0.280	.888
Sleep quality **→** PLEs (WP)	–0.037	0.008	<.001	–0.037	0.008	<.001	–0.018	0.008	.028
Sleep quality **→** PLEs (BP)	–0.152	0.072	.092	–0.133	0.071	.147	–0.040	0.061	.776
Sleep duration **→** PLEs (WP)	–0.002	0.004	.619	–0.002	0.004	.695	0.003	0.004	.776
Sleep duration **→** PLEs (BP)	–0.038	0.046	.619	–0.029	0.046	.695	–0.016	0.038	.776

Unstandardized fixed-effect coefficients (b) and FDR-adjusted *P*-values are reported.

Adjustment 1 includes age, sex, and education as covariates. Adjustment 2 additionally includes negative affect decomposed to within-person and between-person components.

No significant within-person association between sleep duration and PLEs were found. Moreover, none of the tested between-person associations between sleep outcomes and PLEs were significant.

### Effects of Daily Stress

Across all stress categories, within-person fluctuations in daily stress were not significantly associated with next-morning sleep quality or sleep duration, either in unadjusted models or after adjustment for age, sex, education, and negative affect (all *P*-values >.05, Table S3). In contrast, between-person differences in stress exposure were consistently associated with poorer sleep quality, but not sleep duration. Higher average levels of event-related, area-related, activity-related, and social stress were associated with lower sleep quality in unadjusted analyses (all *P*-values <.001). These associations remained significant after adjustment for age, sex, and education, and were only partially attenuated after further adjustment for within- and between-person negative affect (event-related stress: b = −0.622, SE = 0.152, *P* < .001; area-related stress: b = −0.391, SE = 0.137, *P* = .005; activity-related stress: b = −0.582, SE = 0.164, *P* < .001; social stress: b = −0.382, SE = 0.108, *P* < .001). No consistent associations were observed between between-person stress levels and sleep duration (all *P*-values >.050).

Importantly, no significant group × stress interactions were found for either within-person or between-person stress effects across any stress domain or sleep outcome (all FDR-adjusted *P*-values >.050), indicating that associations between daily stress and sleep did not differ between individuals with PLEs and controls. Power simulations revealed that the sample size and the number of observations were sensitive to detecting small-to-moderate group × stress interaction effects (regression coefficients of approximately 0.18-0.20) with 80% power.

### Effects of Childhood Trauma

Associations between childhood trauma and person-level mean and variability of sleep quality and sleep duration are presented in Tables S4 and S5. No significant main effects of childhood trauma history on sleep outcomes were observed. Also, sleep parameters were not associated with the group (participants with PLEs vs. controls) by childhood trauma interactions. For person-level analyses examining childhood trauma, the available sample size provided approximately 80% power to detect trauma-related effects of approximately *f*^2^ = 0.05 or larger in multiple regression models.

## Discussion

### Main Findings

In this ESM study, we examined sleep characteristics, daily stress, and childhood trauma in relation to PLEs in daily life. Individuals with PLEs showed poorer average sleep quality, shorter sleep duration, and greater variability in sleep quality compared with controls. However, differences in sleep quality and its variability were largely attenuated after accounting for negative affect. In participants with PLEs, sleep quality—but not sleep duration—exhibited robust bidirectional associations with PLEs at the within-person level, which remained significant after adjustment for momentary negative affect. In contrast, daily fluctuations in stress were not associated with next-morning sleep outcomes; instead, between-person differences in stress exposure were consistently related to poorer average sleep quality, with no evidence for moderation by group status (individuals with PLEs vs. controls). Finally, childhood trauma was not significantly associated with sleep outcomes in the overall sample, nor did these associations differ between individuals with PLEs and controls.

The finding that individuals with PLEs reported shorter sleep duration than controls, even after accounting for negative affect, suggests that reduced sleep time may represent a relatively stable feature of psychosis-spectrum vulnerability rather than a secondary correlate of concurrent emotional distress. This interpretation is consistent with epidemiological and longitudinal studies linking insomnia to increased risk of psychotic experiences, independent of depressive, and anxiety symptoms.^23^^,^^31^^,^^62^ From a developmental and neurobiological perspective, chronic sleep restriction may contribute to cumulative impairments in cognitive control, emotional regulation, and stress resilience, processes that are centrally implicated in psychosis risk.^63^^,^^64^ Notably, the absence of within-person associations between sleep duration and next-day PLEs in the present study suggests that sleep duration may primarily differentiate individuals with and without PLEs at the between-person level, rather than functioning as a proximal trigger of symptom fluctuations in daily life. This dissociation between stable and dynamic sleep parameters highlights the importance of distinguishing trait-like vulnerability markers from processes involved in momentary symptom expression.

In contrast to sleep duration, sleep quality showed robust bidirectional associations with PLEs at the within-person level among individuals with PLEs, even after adjustment for momentary negative affect. This pattern suggests that perceived sleep quality is closely intertwined with momentary changes in PLEs and may reflect dynamic alterations in affective regulation, arousal, and cognitive processing. Poor sleep quality may increase vulnerability to anomalous perceptions or beliefs by amplifying emotional reactivity and reducing top-down cognitive control, mechanisms that have been implicated in both insomnia and psychosis-spectrum phenomena.^31^^,^^65^ Conversely, heightened PLEs may disrupt subsequent sleep through increased threat anticipation, hyperarousal, or intrusive cognitions, consistent with models emphasizing reciprocal feedback loops between sleep disturbance and psychopathology.^65^^,^^66^ The specificity of these effects to sleep quality rather than sleep duration aligns with prior work highlighting the clinical relevance of subjective sleep complaints and supports the use of intensive longitudinal approaches to capture these dynamic, reciprocal processes in daily life.^67–69^

Contrary to expectations derived from stress-reactivity models,^14^^,^^16^^,^^37^^,^^38^^,^^40^ momentary fluctuations in daily stress were not associated with next-morning sleep outcomes in the present study. Instead, higher between-person levels of stress exposure were consistently related to poorer average sleep quality, suggesting that chronic or trait-like stress burden may be more relevant for sleep disturbances than acute stress reactivity. This pattern aligns with theoretical models of psychosis emphasizing cumulative stress exposure rather than isolated stressors.^16^ A different pattern emerged for childhood trauma, which was not significantly associated with sleep outcomes in the present analyses. Although early adversity has been linked to both sleep disturbances^42^^,^^43^ and psychosis-spectrum vulnerability^70^^,^^71^ in prior research, our findings suggest that sleep dynamics in daily life may be more strongly shaped by proximal regulatory processes rather than distal developmental exposures such as childhood trauma. Such instability may represent a key pathway linking contextual risk processes to psychosis-spectrum experiences.

### Limitations

Certain limitations should be considered when interpreting the findings. First, the study sample consisted predominantly of women. This imbalance was also evident in the broader population approached for participation and likely reflects the greater willingness of women to engage in internet-based research.^72^ Second, the sample did not include individuals with a diagnosed psychotic disorder and participants with a lifetime history of psychiatric treatment were excluded. This design was intended to focus on early manifestations of psychosis-spectrum vulnerability and to minimize potential confounding effects of psychiatric medications on sleep. However, this approach may limit the generalizability of the findings to help-seeking or clinical populations. Third, we did not stratify participants according to distress associated with PLEs, which may provide additional prognostic information in studies focused on clinical transition risk.^10^^,^^73^ Finally, although participants in both groups were recruited within the same age range (18-35 years), individuals reporting PLEs were younger on average than controls. This age difference may reflect the higher prevalence of PLEs in younger individuals rather than a sampling artifact.^74^^,^^75^

In addition, some measurement and design limitations warrant consideration. Although the ESM items employed in this study have been used in prior research,^37^^,^^76–78^ they have not undergone formal psychometric validation. While multi-item scales demonstrated acceptable-to-excellent internal consistency, other aspects of validity, such as factorial, convergent, and discriminant validity, were not evaluated. A further limitation is the reliance on a relatively short ESM protocol and a limited number of self-reported sleep measures rather than objective assessments such as actigraphy or polysomnography. Sleep quality and duration were assessed using brief daily items to limit participant burden and preserve compliance within an intensive sampling protocol. This approach is common in ESM research, where brevity is often necessary to balance measurement precision against feasibility and ecological validity.^29^^,^^31^^,^^77^^,^^78^ Subjective sleep reports may be influenced by perceptual or affective biases, particularly in individuals reporting PLEs. However, self-reported sleep quality and duration are highly relevant to the present research questions, as they capture lived experience of sleep in daily life and are closely linked to affective functioning and psychological distress. Moreover, discrepancies between subjective and objective sleep parameters are common and may themselves reflect meaningful aspects of sleep-related dysregulation, particularly in populations characterized by altered perception and emotional reactivity.^79^^,^^80^

Furthermore, although the study was adequately powered to detect small-to-moderate interaction effects of group status with daily-life stress and childhood trauma history, smaller effects may not have been detected. Also, the present analyses did not test higher-order interaction models integrating PLEs, stress, trauma, and negative affect simultaneously. Such models would require testing multiple higher-order interactions (eg, stress × trauma × PLE status), which may be statistically unstable. Finally, given the observational nature of the study, conclusions regarding causal relationships between sleep, stress, and PLEs should be drawn with caution.

### Clinical Implications

The present findings have several implications for the assessment and early intervention of individuals reporting PLEs. First, the observation that shorter sleep duration in individuals with PLEs remained evident after adjustment for negative affect suggests that reduced sleep time may represent a relatively stable and clinically relevant feature rather than a secondary correlate of mood disturbance. This aligns with evidence showing that insomnia is independently associated with psychosis-spectrum symptoms and broader mental health risk.^62^^,^^81^ Clinically, these findings underscore the importance of routinely assessing sleep duration in individuals presenting with PLEs, even when affective symptoms are not prominent.

Second, the robust bidirectional within-person associations between sleep quality and PLEs highlight sleep quality as a dynamic process closely linked to momentary symptom expression. This finding is consistent with prior studies demonstrating reciprocal associations between subjective sleep disturbance, negative affect, and PLEs or psychotic symptoms.^31–34^ From a clinical perspective, this supports the relevance of interventions targeting perceived sleep quality, such as cognitive–behavioral therapy for insomnia, which has been shown to reduce psychotic experiences alongside improvements in sleep.^81^

Third, the absence of within-person effects of daily stress on next-morning sleep, combined with consistent between-person associations between higher stress exposure and poorer average sleep quality, suggests that trait-like stress burden may be more clinically salient than acute stress reactivity. This pattern is in line with stress-vulnerability models of psychosis emphasizing chronic stress exposure over momentary stressors.^16^^,^^82^ Interventions aimed at reducing ongoing psychosocial stress and enhancing long-term coping resources may therefore have greater impact on sleep quality than strategies focused solely on daily stress management.

Finally, the absence of significant associations between childhood trauma and sleep outcomes in the present analyses suggests that sleep disturbances in daily life may be more closely linked to proximal processes, such as current stress exposure and momentary symptom dynamics, than to distal developmental risk factors. Clinically, this highlights the importance of focusing on modifiable daily-life mechanisms when addressing sleep disturbances in individuals reporting PLEs.

### Conclusions

In summary, this ESM study demonstrates that sleep disturbances are a salient feature of PLEs in daily life, with distinct roles for sleep duration, sleep quality, and sleep variability. Shorter sleep duration emerged as a relatively stable characteristic of individuals with PLEs that was not explained by negative affect, whereas sleep quality showed dynamic, bidirectional associations with momentary PLEs expression. In contrast, daily stress did not exert acute within-person effects on subsequent sleep, but higher between-person stress exposure was consistently linked to poorer average sleep quality. Childhood trauma was not significantly associated with sleep outcomes in the present study. Together, these findings underscore the importance of distinguishing between stable and dynamic components of sleep disturbance and highlight sleep quality and variability as potential markers of vulnerability in the psychosis spectrum. Integrating intensive longitudinal assessments of sleep with developmental and contextual risk factors may advance early identification and inform targeted, transdiagnostic interventions aimed at reducing psychosis-related risk.

## Supplementary Material

Supplementary_Appendix_sbag104

## Data Availability

The data supporting findings from the present study are available in the Open Science Framework (OSF) repository at https://osf.io/8ju6b (file name: “ESM_sleep”, doi:10.17605/OSF.IO/8JU6B).
