# Supplementary material for: Stress and Sleep Dynamics in Young Adults with Psychotic-Like Experiences Across Daily Life
Source: Schizophr Bull. 2026 Aug 29;52(5):sbag104. doi: 10.1093/schbul/sbag104 (PMC13525551; doi:10.1093/schbul/sbag104)
Supplement: Supplementary file 1 [file supplementary_appendix_sbag104.docx]

**Supplementary Appendix for**

Stress and sleep dynamics in young adults with psychotic-like experiences across daily life

Błażej Misiak^1*^, Paulina Bagrowska^2^, Jerzy Samochowiec^3^, Łukasz Gawęda^2^

^1^Department of Psychiatry, Wroclaw Medical University, Wroclaw, Poland

^2^Experimental Psychopathology Lab, Institute of Psychology, Polish Academy of Sciences, Warsaw, Poland

^3^Department of Psychiatry, Pomeranian Medical University, Szczecin, Poland

^*^Corresponding author

e-mail: [blazej.misiak@umw.edu.pl](mailto:blazej.misiak@umw.edu.pl)

**Contents**

**Figure S1.** Flow diagram of participants ………………………………………………………………………………………………………………2

**Table S1.** ESM items ……………………………………………………………………………………………………………………………………………3

**Table S2.** Dimensional between-person associations between mean levels of psychotic-like experiences (PLEs) and person-level sleep parameters in participants with PLEs………………………………………………………………………………4

**Table S3.** Associations of daily stress with next-morning sleep quality and duration in participants with and without psychotic-like experiences …………………………………………………………………………………………………………………….5

**Table S4.** Associations of childhood trauma history with mean sleep outcomes………………………………………………….7

**Table S5.** Associations of childhood trauma history with variability of sleep outcomes……………………………………….8

**Figure S1.** Flow diagram of participants.

**Table S1.** The ESM items.

| Domain | Items | Cronbach’s alpha | |
| --- | --- | --- | --- |
|  |  | Within-subjects | Between-subjects |
| Sleep quality | “How would you rate your sleep last night?” | – | – |
| Sleep duration | “How many hours did you sleep last night (time from falling asleep to waking up, in hours)? | – | – |
| Activity-related stress | “I would prefer doing something else”  “My current activity is difficult to me”  “My current activity is pleasant to me” (reversed scoring) | 0.78 | 0.80 |
| Social stress | “Whom are you staying with right now?”  “I would prefer to be alone (if with someone)/I would prefer to have company (if alone)”  “I find being with these people pleasant (if with someone)/it is pleasant to be alone (if alone)” (reversed scoring) | 0.76 | 0.81 |
| Event-related stress | “Think about the most important event since the last beep (“during the last two hours” in case of the first beep during specific day). This event was…”. | – | – |
| Area-related stress | “I feel being in this neighborhood unpleasant” | – | – |
| Negative affect | “I feel anxious”  “I feel down”  “I feel insecure”  “I feel annoyed” | 0.74 | 0.92 |
| Psychotic-like experiences | “My thoughts are so strong that I can almost hear them”  “I hear things that aren’t really there”  “I see things that aren’t really there”  “I have the sense that some person or force is around me although I can’t see anyone”  “I see special meanings in advertisements, shop windows, or in the way things are arranged around me”  “I am confused whether something I experienced was real or imaginary”,  “My thoughts are influenced by others”  “I can’t get these thoughts out of my head” | 0.72 | 0.90 |

Items were rated on a 7-point scale: 1 – “not at all” (for sleep quality: “very bad”; for event-related stress: 1 – “very unpleasant”) and 7 – “very much” (for sleep quality: “very good”; for event-related stress: “very pleasant”). For sleep duration, participants were asked to provide the number of hours.

**Table S2.** Dimensional between-person associations between mean levels of psychotic-like experiences (PLEs) and person-level sleep parameters in participants with PLEs.

| Sleep outcome | Unadjusted analysis | Adjustment 1 | Adjustment 2 |
| --- | --- | --- | --- |
| Mean sleep duration | b = –0.160,  SE = 0.220,  p = 0.469 | b = –0.119,  SE = 0.227,  p = 0.601 | b = –0.088,  SE = 0.277,  p = 0.751 |
| Mean sleep quality | b = –0.286,  SE = 0.136,  p = 0.075 | b = –0.258,  SE = 0.141,  p = 0.141 | b = –0.108,  SE = 0.171,  p = 0.751 |
| Sleep duration variability | b = 0.333,  SE = 0.382,  p = 0.385 | b = 0.352,  SE = 0.397,  p = 0.378 | b = 0.301,  SE = 0.486,  p = 0.537 |
| Sleep quality variability | b = 0.128,  SE = 0.071,  p = 0.151 | b = 0.111,  SE = 0.071,  p = 0.240 | b = 0.132,  SE = 0.086,  p = 0.259 |

Adjustment 1 includes the effects of age, sex, and the level of education. Adjustment 2 includes the effects of age, sex, the level of education, and mean negative affect. Unstandardized coefficients (b) and FDR-adjusted p-values are reported

**Table S3.** Associations of daily stress with next-morning sleep quality and duration in participants with and without psychotic-like experiences.

| Stress category | Effect | Unadjusted analysis | | Adjustment 1 | | Adjustment 2 | |
| --- | --- | --- | --- | --- | --- | --- | --- |
|  |  | Sleep quality | Sleep duration | Sleep quality | Sleep duration | Sleep quality | Sleep duration |
| Event-related stress | Group | b = –0.348,  SE = 0.745,  p = 0.641 | b = 1.850,  SE = 1.286,  p = 0.152 | b = –0.334  SE = 0.752,  p = 0.657 | b = 1.379,  SE = 1.323,  p = 0.299 | b = –0.309,  SE = 0.746,  p = 0.679 | b = 1.337,  SE = 1.320,  p = 0.312 |
|  | Stress (WP) | b = –0.009,  SE = 0.095,  p = 0.926 | b = –0.187,  SE = 0.235,  p = 0.427 | b = –0.027,  SE = 0.097,  p = 0.781 | b = –0.199,  SE = 0.244,  p = 0.414 | b = –0.006,  SE = 0.099,  p = 0.955 | b = –0.125,  SE = 0.250,  p = 0.617 |
|  | Stress (BP) | b = –0.693,  SE = 0.148,  p < 0.001 | b = 0.345,  SE = 0.256,  p = 0.180 | b = –0.684,  SE = 0.149,  p < 0.001 | b = 0.310,  SE = 0.262,  p = 0.238 | b = –0.622,  SE = 0.152,  p < 0.001 | b = 0.255,  SE = 0.269,  p = 0.345 |
|  | Group × stress (WP) | b = 0.097,  SE = 0.122,  p = 0.743 | b = –0.187,  SE = 0.235,  p = 0.434 | b = 0.132,  SE = 0.125,  p = 0.696 | b = 0.599,  SE = 0.314,  p = 0.454 | b = 0.162,  SE = 0.206  p = 0.647 | b = 0.694,  SE = 0.322,  p = 0.253 |
|  | Group × stress (BP) | b = –0.040,  SE = 0.213,  p = 0.853 | b = –0.698,  SE = 0.368,  p = 0.326 | b = –0.040,  SE = 0.215,  p = 0.853 | b = –0.568,  SE = 0.379,  p = 0.436 | b = 0.001,  SE = 0.215,  p = 0.999 | b = –0.599,  SE = 0.380,  p = 0.312 |
| Area-related stress | Group | b = –1.173,  SE = 0.402,  p = 0.004 | b = –0.017,  SE = 0.661,  p = 0.979 | b = –1.021,  SE = 0.420,  p = 0.016 | b = –0.161,  SE = 0.708,  p = 0.820 | b = –0.975,  SE = 0.415,  p = 0.020 | b = –0.196,  SE = 0.708,  p = 0.782 |
|  | Stress (WP) | b = –0.101,  SE = 0.079,  p = 0.203 | b = –0.034,  SE = 0.196,  p = 0.864 | b = –0.097,  SE = 0.083,  p = 0.242 | b = –0.036,  SE = 0.207,  p = 0.861 | b = –0.110,  SE = 0.085,  p = 0.195 | b = –0.002,  SE = 0.212,  p = 0.992 |
|  | Stress (BP) | b = –0.493,  SE = 0.131,  p < 0.001 | b = 0.096,  SE = 0.215,  p = 0.656 | b = –0.488,  SE = 0.132,  p < 0.001 | b = 0.130,  SE = 0.222,  p = 0.559 | b = –0.391,  SE = 0.137,  p = 0.005 | b = 0.065,  SE = 0.233,  p = 0.779 |
|  | Group × stress (WP) | b = 0.006,  SE = 0.106,  p = 0.955 | b = 0.178,  SE = 0.262,  p = 0.955 | b = 0.023,  SE = 0.111,  p = 0.925 | b = 0.232,  SE = 0.278,  p = 0.696 | b = 0.005,  SE = 0.113,  p = 0.967 | b = 0.279,  SE = 0.285,  p = 0.647 |
|  | Group × stress (BP) | b = 0.292,  SE = 0.166,  p = 0.326 | b = –0.225,  SE = 0.274,  p = 0.326 | b = 0.241,  SE = 0.172,  p = 0.436 | b = –0.184,  SE = 0.290,  p = 0.767 | b = 0.320,  SE = 0.173,  p = 0.312 | b = –0.237,  SE = 0.296,  p = 0.751 |
| Activity-related stress | Group | b = –1.319,  SE = 0.641,  p = 0.041 | b = –1.101,  SE = 1.084,  p = 0.311 | b = –1.280,  SE = 0.655,  p = 0.052 | b = –1.190,  SE = 1.141,  p = 0.298 | b = –1.282,  SE = 0.653,  p = 0.051 | b = –1.190,  SE = 1.139,  p = 0.297 |
|  | Stress (WP) | b = –0.076,  SE = 0.086,  p = 0.381 | b = 0.034,  SE = 0.214,  p = 0.873 | b = –0.070,  SE = 0.089,  p = 0.427 | b = 0.060,  SE = 0.223,  p = 0.788 | b = –0.067,  SE = 0.093,  p = 0.467 | b = 0.127,  SE = 0.233,  p = 0.585 |
|  | Stress (BP) | b = –0.666,  SE = 0.144,  p < 0.001 | b = –0.101,  SE = 0.243,  p = 0.679 | b = –0.672,  SE = 0.149,  p < 0.001 | b = –0.043,  SE = 0.259,  p = 0.868 | b = –0.582,  SE = 0.164,  p < 0.001 | b = –0.113,  SE = 0.286,  p = 0.693 |
|  | Group × stress (WP) | b = 0.080,  SE = 0.111,  p = 0.743 | b = 0.162,  SE = 0.276,  p = 0.743 | b = 0.089,  SE = 0.114,  p = 0.696 | b = 0.145,  SE = 0.287,  p = 0.817 | b = 0.090,  SE = 0.117,  p = 0.647 | b = 0.204,  SE = 0.293,  p = 0.647 |
|  | Group × stress (BP) | b = 0.297,  SE = 0.197,  p = 0.357 | b = –0.101  SE = 0.334,  p = 0.734 | b = 0.289,  SE = 0.201,  p = 0.436 | b = 0.186,  SE = 0.350,  p = 0.767 | b = 0.324,  SE = 0.202,  p = 0.312 | b = 0.158,  SE = 0.352,  p = 0.751 |
| Social stress | Group | b = –0.745,  SE = 0.437,  p = 0.089 | b = –0.877,  SE = 0.751,  p = 0.245 | b = –0.722,  SE = 0.437,  p = 0.100 | b = –0.859,  SE = 0.753,  p = 0.256 | b = 0.296,  SE = 0.441,  p = 0.296 | b = –1.190,  SE = 1.139,  p = 0.297 |
|  | Stress (WP) | b = –0.029,  SE = 0.075,  p = 0.704 | b = 0.082,  SE = 0.188,  p = 0.663 | b = –0.028,  SE = 0.075,  p = 0.712 | b = 0.089,  SE = 0.189,  p = 0.638 | b = –0.029,  SE = 0.077,  p = 0.704 | b = –0.127,  SE = 0.233,  p = 0.585 |
|  | Stress (BP) | b = –0.420,  SE = 0.108,  p < 0.001 | b = –0.083,  SE = 0.187,  p = 0.663 | b = 0.442,  SE = 0.107,  p < 0.001 | b = –0.072,  SE = 0.186,  p = 0.701 | b = –0.382,  SE = 0.108,  p < 0.001 | b = –0.113,  SE = 0.286,  p = 0.693 |
|  | Group × stress (WP) | b = 0.009,  SE = 0.094,  p = 0.955 | b = 0.204,  SE = 0.236,  p = 0.743 | b = 0.009,  SE = 0.094,  p = 0.925 | b = 0.200,  SE = 0.236,  p = 0.696 | b = 0.011,  SE = 0.094,  p = 0.967 | b = 0.204,  SE = 0.293,  p = 0.647 |
|  | Group × stress (BP) | b = 0.083,  SE = 0.147,  p = 0.734 | b = 0.118,  SE = 0.254,  p = 0.734 | b = 0.101,  SE = 0.146,  p = 0.767 | b = 0.107,  SE = 0.252,  p = 0.767 | b = 0.098,  SE = 0.144,  p = 0.751 | b = 0.158,  SE = 0.352,  p = 0.751 |

Adjustment 1 includes the effects of age, sex, and the level of education. Adjustment 2 includes the effects of age, sex, the level of education, within- and between-person effects of negative affect. Unstandardized coefficients (b) are reported. Group status refers to the comparison of participants with psychotic-like experiences vs. controls. P-values for interaction effects are FDR-adjusted.

*Note:* BP, between-person effects; WP, within-person effects

**Table S4.** Associations of childhood trauma history with mean sleep outcomes.

| Trauma category | Effect | Unadjusted analysis | | Adjustment 1 | | Adjustment 2 | |
| --- | --- | --- | --- | --- | --- | --- | --- |
|  |  | Sleep quality | Sleep duration | Sleep quality | Sleep duration | Sleep quality | Sleep duration |
| Emotional neglect | Group | b = –0.701,  SE = 0.147,  p < 0.001 | b = –0.638,  SE = 0.238,  p = 0.008 | b = –0.651,  SE = 0.150,  p < 0.001 | b = –0.617,  SE = 0.244,  p = 0.012 | b = –0.181,  SE = 0.174,  p = 0.301 | b = –0.759,  SE = 0.301,  p = 0.013 |
|  | Trauma | b = –0.010,  SE = 0.023,  p = 0.941 | b = –0.003,  SE = 0.038,  p = 0.943 | b = 0.001,  SE = 0.024,  p = 0.971 | b = –0.006,  SE = 0.039,  p = 0.971 | b = –0.002,  SE = 0.023,  p= 0.976 | b = –0.005,  SE = 0.039,  p = 0.976 |
|  | Group × trauma | b = 0.010,  SE = 0.034,  p = 0.978 | b = 0.061,  SE = 0.056,  p = 0.277 | b = 0.017,  SE = 0.035,  p = 0.904 | b = 0.069,  SE = 0.057,  p = 0.797 | b = 0.028,  SE = 0.033,  p = 0.794 | b = 0.066,  SE = 0.057,  p = 0.794 |
| Emotional abuse | Group | b = –0.716,  SE = 0.156,  p < 0.001 | b = –0.466,  SE = 0.252,  p = 0.066 | b = –0.661,  SE = 0.158,  p < 0.001 | b = –0.481,  SE = 0.259,  p = 0.067 | b = –0.193,  SE = 0.182,  p = 0.291 | b = –0.644,  SE = 0.315,  p = 0.042 |
|  | Trauma | b = 0.013,  SE = 0.030,  p = 0.941 | b = –0.033,  SE = 0.049,  p = 0.941 | b = 0.019,  SE = 0.030,  p = 0.971 | b = –0.026,  SE = 0.049,  p = 0.971 | b = 0.013,  SE = 0.029,  p = 0.976 | b = –0.024,  SE = 0.049,  p = 0.976 |
|  | Group × trauma | b = –0.022,  SE = 0.041,  p = 0.947 | b = –0.006,  SE = 0.066,  p = 0.978 | b = –0.020,  SE = 0.041,  p = 0.904 | b = –0.014,  SE = 0.067,  p = 0.933 | b = –0.012,  SE = 0.039,  p = 0.911 | b = –0.017,  SE = 0.067,  p = 0.911 |
| Physical abuse | Group | b = –0.690,  SE = 0.142,  p < 0.001 | b = –0.529,  SE = 0.231,  p = 0.023 | b = –0.630,  SE = 0.147,  p < 0.001 | b = –0.548,  SE = 0.240,  p = 0.024 | b = –0.165,  SE = 0.172,  p = 0.341 | b = –0.712,  SE = 0.298,  p = 0.018 |
|  | Trauma | b = –0.029,  SE = 0.050,  p = 0.941 | b = –0.029,  SE = 0.050,  p = 0.941 | b = 0.008,  SE = 0.031,  p = 0.971 | b = –0.021,  SE = 0.050,  p = 0.681 | b = 0.011,  SE = 0.029,  p = 0.976 | b = –0.022,  SE = 0.050,  p = 0.976 |
|  | Group × trauma | b = –0.066,  SE = 0.043,  p = 0.810 | b = –0.065,  SE = 0.070,  p = 0.810 | b = –0.055,  SE = 0.044,  p = 0.797 | b = –0.074,  SE = 0.072,  p = 0.797 | b = –0.058,  SE = 0.042,  p = 0.794 | b = –0.073,  SE = 0.072,  p = 0.794 |
| Sexual abuse | Group | b = –0.625,  SE = 0.151,  p < 0.001 | b = –0.513,  SE = 0.246,  p = 0.038 | b = –0.591,  SE = 0.155,  p < 0.001 | b = –0.521,  SE = 0.252,  p = 0.041 | b = –0.146,  SE = 0.177,  p = 0.410 | b = –0.671,  SE = 0.303,  p = 0.028 |
|  | Trauma | b = –0.115,  SE = 0.125,  p = 0.941 | b = 0.044,  SE = 0.203,  p = 0.941 | b = –0.082,  SE = 0.126,  p = 0.971 | b = 0.014,  SE = 0.204,  p = 0.971 | b = –0.022,  SE = 0.120,  p = 0.976 | b = –0.006,  SE = 0.206,  p = 0.976 |
|  | Group × trauma | b = 0.014,  SE = 0.139,  p = 0.978 | b = –0.239,  SE = 0.226,  p = 0.810 | b = 0.022,  SE = 0.139,  p = 0.933 | b = –0.230,  SE = 0.225,  p = 0.797 | b = –0.042,  SE = 0.132,  p = 0.911 | b = –0.208,  SE = 0.227,  p = 0.794 |

Adjustment 1 includes the effects of age, sex, and the level of education. Adjustment 2 includes the effects of age, sex, the level of education, within- and between-person effects of negative affect. Unstandardized coefficients (b) are reported. Group status refers to the comparison of participants with psychotic-like experiences vs. controls. P-values for main effects of childhood trauma and interaction effects are FDR-adjusted.

**Table S5.** Associations of childhood trauma history with variability of sleep outcomes.

| Trauma category | Effect | Unadjusted analysis | | Adjustment 1 | | Adjustment 2 | |
| --- | --- | --- | --- | --- | --- | --- | --- |
|  |  | Sleep quality | Sleep duration | Sleep quality | Sleep duration | Sleep quality | Sleep duration |
| Emotional neglect | Group | b = 0.169,  SE = 0.077,  p = 0.030 | b = 0.248,  SE = 0.452,  p = 0.941 | b = 0.166,  SE = 0.077,  p = 0.033 | b = 0.171,  SE = 0.468,  p = 0.715 | b = 0.104,  SE = 0.095,  p = 0.277 | b = –0.325,  SE = 0.574,  p = 0.572 |
|  | Trauma | b = 0.002,  SE = 0.012,  p = 0.941 | b = –0.055,  SE = 0.072,  p = 0.444 | b = –0.073,  SE = 0.075,  p = 0.971 | b = –0.072,  SE = 0.075,  p = 0.971 | b = –0.003,  SE = 0.012,  p = 0.976 | b = –0.070,  SE = 0.074,  p = 0.976 |
|  | Group × trauma | b = 0.012,  SE = 0.018,  p = 0.921 | b = 0.119,  SE = 0.106,  p = 0.810 | b = –0.028,  SE = 0.021,  p = 0.797 | b = 0.124,  SE = 0.108,  p = 0.255 | b = 0.010,  SE = 0.018,  p = 0.911 | b = 0.113,  SE = 0.108,  p = 0.794 |
| Emotional abuse | Group | b = 0.167,  SE = 0.081,  p = 0.041 | b = 0.283,  SE = 0.452,  p = 0.556 | b = 0.156,  SE = 0.081,  p = 0.057 | b = 0.190,  SE = 0.497,  p = 0.702 | b = 0.086,  SE = 0.099,  p = 0.385 | b = –0.005,  SE = 0.601,  p = 0.587 |
|  | Trauma | b = 0.020,  SE = 0.016,  p = 0.941 | b = –0.049,  SE = 0.093,  p = 0.941 | b = –0.044,  SE = 0.094,  p = 0.971 | b = –0.044,  SE = 0.094,  p = 0.971 | b = 0.020,  SE = 0.016,  p = 0.976 | b = –0.038,  SE = 0.094,  p = 0.976 |
|  | Group × trauma | b = –0.027,  SE = 0.021,  p = 0.810 | b = 0.082,  SE = 0.126,  p = 0.921 | b = 0.012,  SE = 0.018,  p = 0.904 | b = 0.067,  SE = 0.128,  p = 0.904 | b = –0.029,  SE = 0.021,  p = 0.794 | b = 0.058,  SE = 0.127,  p = 0.911 |
| Physical abuse | Group | b = 0.165,  SE = 0.074,  p = 0.027 | b = 0.303,  SE = 0.441,  p = 0.493 | b = 0.158,  SE = 0.075,  p = 0.037 | b = 0.189,  SE = 0.463,  p = 0.684 | b = 0.094,  SE = 0.093,  p = 0.314 | b = –0.336,  SE = 0.572,  p = 0.557 |
|  | Trauma | b = 0.017,  SE = 0.016,  p = 0.941 | b = –0.053,  SE = 0.096,  p = 0.941 | b = –0.044,  SE = 0.097,  p = 0.971 | b = –0.044,  SE = 0.097,  p = 0.971 | b = 0.016,  SE = 0.016,  p = 0.976 | b = –0.047,  SE = 0.096,  p = 0.976 |
|  | Group × trauma | b = 0.022,  SE = 0.022,  p = 0.810 | b = –0.004,  SE = 0.133,  p = 0.978 | b = 0.021,  SE = 0.022,  p = 0.797 | b = –0.026,  SE = 0.138,  p = 0.933 | b = 0.021,  SE = 0.022,  p = 0.794 | b = –0.022,  SE = 0.138,  p = 0.911 |
| Sexual abuse | Group | b = 0.117,  SE = 0.077,  p = 0.134 | b = 0.345,  SE = 0.470,  p = 0.464 | b = 0.128,  SE = 0.079,  p = 0.104 | b = 0.257,  SE = 0.486,  p = 0.597 | b = 0.068,  SE = 0.095,  p = 0.474 | b = –0.257,  SE = 0.583,  p = 0.660 |
|  | Trauma | b = 0.074,  SE = 0.064,  p = 0.941 | b = –0.089,  SE = 0.389,  p = 0.941 | b = –0.143,  SE = 0.394,  p = 0.971 | b = –0.143,  SE = 0.394,  p = 0.971 | b = 0.049,  SE = 0.064,  p = 0.976 | b = –0.213,  SE = 0.395,  p = 0.976 |
|  | Group × trauma | b = 0.024,  SE = 0.072,  p = 0.978 | b = –0.030,  SE = 0.433,  p = 0.945 | b = 0.024,  SE = 0.070,  p = 0.933 | b = –0.024,  SE = 0.435,  p = 0.955 | b = 0.033,  SE = 0.071,  p = 0.911 | b = 0.049,  SE = 0.436,  p = 0.911 |

Adjustment 1 includes the effects of age, sex, and the level of education. Adjustment 2 includes the effects of age, sex, the level of education, within- and between-person effects of negative affect. Unstandardized coefficients (b) are reported. Group status refers to the comparison of participants with psychotic-like experiences vs. controls. P-values for main effects of childhood trauma and interaction effects are FDR-adjusted.
